# Diaqua­bis(*N*,*N*-diethyl­nicotinamide-κ*N* ^1^)bis­(4-formyl­benzoato-κ*O* ^1^)manganese(II)

**DOI:** 10.1107/S1600536809006047

**Published:** 2009-02-25

**Authors:** Mustafa Sertçelik, Barış Tercan, Ertan Şahin, Hacali Necefoğlu, Tuncer Hökelek

**Affiliations:** a Department of Chemistry, Kafkas University, 63100 Kars, Turkey; b Department of Physics, Karabük University, 78050 Karabük, Turkey; c Department of Chemistry, Atatürk University, 22240 Erzurum, Turkey; d Department of Physics, Hacettepe University, 06800 Beytepe, Ankara, Turkey

## Abstract

The title compound, [Mn(C_8_H_5_O_3_)_2_(C_10_H_14_N_2_O)_2_(H_2_O)_2_], contains one Mn^II^ atom lying on an inversion centre, two 4-formyl­benzoate and two diethyl­nicotinamide ligands and two coordinated water mol­ecules. All ligands are monodentate. The four O atoms around the Mn atom form a slightly distorted equatorial plane, while the distorted octa­hedral coordination is completed by the two N atoms in the axial positions. An intra­molecular O—H⋯O hydrogen bond occurs in the complex. In the crystal structure, O—H⋯O hydrogen bonds link the mol­ecules through an *R*_2_^2^(16) ring motif, forming a one-dimensional chain along the *a* axis. The π–π contact between the pyridyl rings [centroid–centroid distance = 3.629 (2) Å] may further stabilize the structure.

## Related literature

For general background, see: Antolini *et al.* (1982 ▶); Nadzhafov *et al.* (1981 ▶); Shnulin *et al.* (1981 ▶). For related structures, see: Hökelek *et al.* (1995 ▶, 1997 ▶, 2007 ▶, 2008 ▶); Hökelek & Necefoğlu (1996 ▶, 1997 ▶, 2007 ▶). For hydrogen-bonding motifs, see: Bernstein *et al.* (1995 ▶).
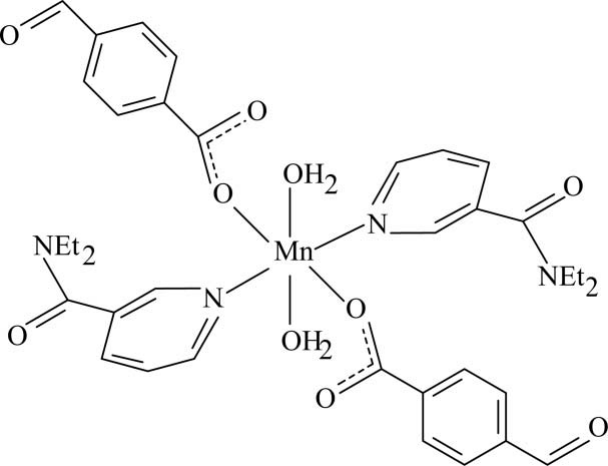


## Experimental

### 

#### Crystal data

[Mn(C_8_H_5_O_3_)_2_(C_10_H_14_N_2_O)_2_(H_2_O)_2_]
*M* *_r_* = 745.68
Triclinic, 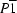

*a* = 7.3266 (2) Å
*b* = 8.6618 (2) Å
*c* = 16.0687 (3) Å
α = 86.381 (8)°
β = 78.272 (7)°
γ = 68.618 (6)°
*V* = 929.67 (6) Å^3^
*Z* = 1
Mo *K*α radiation
μ = 0.42 mm^−1^
*T* = 294 K
0.35 × 0.20 × 0.15 mm

#### Data collection

Rigaku R-AXIS RAPID-S diffractometer
Absorption correction: multi-scan (*ABSCOR*; Higashi, 1995 ▶) *T*_min_ = 0.904, *T*_max_ = 0.935
18356 measured reflections
3799 independent reflections
3317 reflections with *I* > 2σ(*I*)
*R*_int_ = 0.071

#### Refinement

*R*[*F* ^2^ > 2σ(*F* ^2^)] = 0.058
*wR*(*F* ^2^) = 0.157
*S* = 1.02
3799 reflections
246 parameters
3 restraints
H atoms treated by a mixture of independent and constrained refinement
Δρ_max_ = 0.77 e Å^−3^
Δρ_min_ = −0.32 e Å^−3^

### 

Data collection: *PROCESS-AUTO* (Rigaku, 1998 ▶); cell refinement: *PROCESS-AUTO*; data reduction: *PROCESS-AUTO*; program(s) used to solve structure: *SHELXS97* (Sheldrick, 2008 ▶); program(s) used to refine structure: *SHELXL97* (Sheldrick, 2008 ▶); molecular graphics: *ORTEP-3* (Farrugia, 1997 ▶); software used to prepare material for publication: *WinGX* (Farrugia, 1999 ▶).

## Supplementary Material

Crystal structure: contains datablocks I, global. DOI: 10.1107/S1600536809006047/hy2182sup1.cif

Structure factors: contains datablocks I. DOI: 10.1107/S1600536809006047/hy2182Isup2.hkl

Additional supplementary materials:  crystallographic information; 3D view; checkCIF report

## Figures and Tables

**Table 1:** Selected bond lengths (Å)

Mn1—O1	2.1596 (18)
Mn1—O5	2.207 (2)
Mn1—N1	2.283 (2)

**Table 2:** Hydrogen-bond geometry (Å, °)

*D*—H⋯*A*	*D*—H	H⋯*A*	*D*⋯*A*	*D*—H⋯*A*
O5—H51⋯O4^i^	0.92 (3)	1.87 (3)	2.775 (3)	165 (4)
O5—H52⋯O2	0.93 (3)	1.77 (4)	2.669 (3)	162 (4)

Symmetry code: (i) 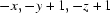.

**Fig. 1.**
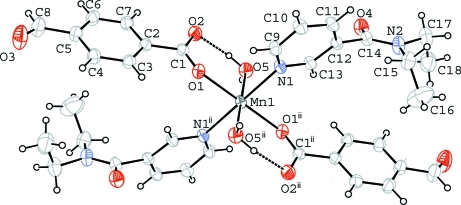
The molecular structure of the title compound. Displacement ellipsoids are drawn at the 40% probability level. Hydrogen bonds are shown as dashed lines. [Symmetry code: (ii) 1 - x, 1 - y, 1 - z.]

**Fig. 2.**
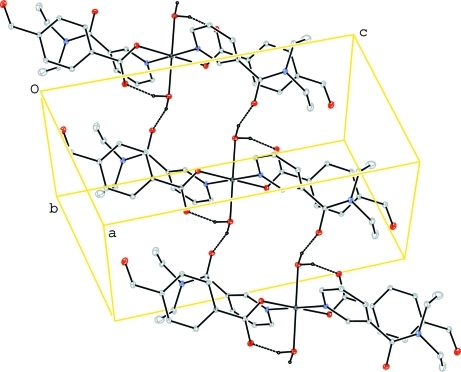
A partial packing diagram of the title compound, showing hydrogen bonds (dashed lines) linking the molecules through the R22(16) ring motif. H atoms not involved in hydrogen bonds are omitted for clarity.

## supplementary crystallographic information

### Comment

The structural functions and coordination relationships of the arylcarboxylates in transition metal complexes of benzoic acid derivatives change depending on the nature and position of the substituent groups on the benzene ring, the nature of the additional ligand molecules or solvents, and the medium of the synthesis (Nadzhafov *et al.*, 1981; Shnulin *et al.*, 1981). Transition metal complexes with biochemically active ligands frequently show interesting physical and/or chemical properties, and as a result, they may find applications in biological systems (Antolini *et al.*, 1982). The structure determination of the title compound, (I), a manganese complex with two formylbenzoate (FOB) and two diethylnicotinamide (DENA) ligands and two water molecules, was undertaken in order to determine the properties of the ligands and also to compare the results obtained with those reported previously.

Compound (I) is a monomeric complex, with the Mn^II^ atom lying on a centre of symmetry. It contains two FOB and two DENA ligands and two water molecules (Fig. 1). All ligands are monodentate. The four O atoms [O1, O5, and the symmetry-related atoms, O1^ii^, O5^ii^; symmetry code: (ii) 1 - *x*, 1 - *y*, 1 - *z*] around the Mn atom form a slightly distorted equatorial plane, while the slightly distorted octahedral coordination is completed by the two N atoms of the DENA ligands (N1, N1^ii^) in the axial positions (Table 1 and Fig. 1). The intramolecular O—H···O hydrogen bond (Table 2) results in the formation of a six-membered ring (Mn1, O1, O2, O5, C1, H52), which adopts envelope conformation with C1 atom displaced by -0.235 (3) Å from the plane of the other five atoms.

The near equality of the C1—O1 [1.262 (3) Å] and C1—O2 [1.249 (3) Å] bonds in the carboxylate group indicates a delocalized bonding arrangement, rather than localized single and double bonds, and may be compared with the corresponding distances: 1.256 (6) and 1.245 (6) Å in [Mn(DENA)_2_(C_7_H_4_ClO_2_)_2_(H_2_O)_2_], (II), (Hökelek *et al.*, 2008), 1.265 (6) and 1.275 (6) Å in [Mn(C_9_H_10_NO_2_)_2_(H_2_O)_4_].2H_2_O, (III), (Hökelek & Necefoğlu, 2007), 1.260 (4) and 1.252 (4) Å in [Zn(DENA)_2_(C_7_H_4_FO_2_)_2_(H_2_O)_2_], (IV), (Hökelek *et al.*, 2007), 1.259 (9) and 1.273 (9) Å in [Cu_2_(DENA)_2_(C_6_H_5_COO)_4_], (V), (Hökelek *et al.*, 1995), 1.279 (4) and 1.246 (4) Å in [Zn_2_(DENA)_2_(C_7_H_5_O_3_)_4_].2H_2_O, (VI), (Hökelek & Necefoğlu, 1996), 1.251 (6) and 1.254 (7) Å in [Co(DENA)_2_(C_7_H_5_O_3_)_2_(H_2_O)_2_], (VII), (Hökelek & Necefoğlu, 1997), 1.278 (3) and 1.246 (3) Å in [Cu(DENA)_2_(C_7_H_4_NO_4_)_2_(H_2_O)_2_], (VIII), (Hökelek *et al.*, 1997). This may be due to the intramolecular O—H···O hydrogen bond involving the carboxylate O atom (Table 2). In (I), the average Mn—O bond length is 2.183 (2) Å and the Mn atom is displaced out of the least-squares plane of the carboxylate group (O1, C1, O2) by -0.859 (1) Å. They are reported as -0.890 (1) Å in (II) and 2.185 (4) and 1.365 (3) Å in (III). The dihedral angle between the planar carboxylate group and the benzene ring A (C2–C7) is 3.0 (2)°, while that between rings A and B (N1, C9–C13) is 80.0 (1)°.

In the crystal structure, intermolecular O—H···O hydrogen bonds (Table 2) link the molecules through a *R*^2^_2_(16) ring motif (Bernstein *et al.*, 1995) to form a one-dimensional chain along the *a* axis (Fig. 2). The π-π contact between the pyridyl rings of DENA ligands, *Cg*1···*Cg*1^iii^ [symmetry code: (iii) -*x*, 2 - *y*, 1 - *z*; where *Cg*1 is the centroid of ring B] may further stabilize the structure, with centroid–centroid distance of 3.629 (2) Å.

### Experimental

The title compound was prepared by the reaction of Mn(SO_4_).H_2_O (1.69 g, 10 mmol) in H_2_O (50 ml) and DENA (3.56 g, 20 mmol) in H_2_O (15 ml) with sodium 4-formylbenzoate (3.44 g, 20 mmol) in H_2_O (50 ml). The mixture was filtered and set aside to crystallize at ambient temperature for several days, giving colourless single crystals.

### Refinement

H atoms of water molecule and formyl group were located on difference Fourier maps and refined isotropically, with restrains of O—H = 0.95 (2) and C—H = 0.96 (2) Å. The remaining H atoms were positioned geometrically and refined as riding atoms, with C—H = 0.93 (aromatic), 0.97 (methylene) and 0.96 (methyl) Å, and with *U*_iso_(H) = *xU*_eq_(C), where *x* = 1.5 for methyl H atoms and 1.2 for the other H atoms.

### Crystal data

**Table:**

[Mn(C_8_H_5_O_3_)_2_(C_10_H_14_N_2_O)_2_(H_2_O)_2_]	*Z* = 1
*M**_r_* = 745.68	*F*(000) = 391
Triclinic, *P*1	*D*_x_ = 1.332 Mg m^−^^3^
Hall symbol: -P 1	Mo *K*α radiation, λ = 0.71073 Å
*a* = 7.3266 (2) Å	Cell parameters from 2942 reflections
*b* = 8.6618 (2) Å	θ = 2.5–26.4°
*c* = 16.0687 (3) Å	µ = 0.42 mm^−^^1^
α = 86.381 (8)°	*T* = 294 K
β = 78.272 (7)°	Block, colourless
γ = 68.618 (6)°	0.35 × 0.20 × 0.15 mm
*V* = 929.67 (6) Å^3^	

### Data collection

**Table:**

Rigaku R-AXIS RAPID-S diffractometer	3799 independent reflections
Radiation source: fine-focus sealed tube	3317 reflections with *I* > 2σ(*I*)
graphite	*R*_int_ = 0.071
ω scans	θ_max_ = 26.4°, θ_min_ = 2.5°
Absorption correction: multi-scan (*ABSCOR*; Higashi, 1995)	*h* = −9→9
*T*_min_ = 0.904, *T*_max_ = 0.935	*k* = −10→10
18356 measured reflections	*l* = −20→20

### Refinement

**Table:**

Refinement on *F*^2^	Primary atom site location: structure-invariant direct methods
Least-squares matrix: full	Secondary atom site location: difference Fourier map
*R*[*F*^2^ > 2σ(*F*^2^)] = 0.058	Hydrogen site location: inferred from neighbouring sites
*wR*(*F*^2^) = 0.157	H atoms treated by a mixture of independent and constrained refinement
*S* = 1.02	*w* = 1/[σ^2^(*F*_o_^2^) + (0.0681*P*)^2^ + 0.7177*P*] where *P* = (*F*_o_^2^ + 2*F*_c_^2^)/3
3799 reflections	(Δ/σ)_max_ < 0.001
246 parameters	Δρ_max_ = 0.77 e Å^−^^3^
3 restraints	Δρ_min_ = −0.32 e Å^−^^3^

### Fractional atomic coordinates and isotropic or equivalent isotropic displacement parameters (Å^2^)

**Table:**

	*x*	*y*	*z*	*U*_iso_*/*U*_eq_	
Mn1	0.5000	0.5000	0.5000	0.04035 (19)	
O1	0.5226 (3)	0.6203 (2)	0.60914 (12)	0.0488 (5)	
O2	0.2455 (3)	0.6315 (3)	0.70146 (14)	0.0583 (5)	
O3	0.9452 (5)	0.6893 (5)	0.95632 (19)	0.1013 (11)	
O4	−0.2292 (3)	0.8295 (3)	0.37375 (14)	0.0615 (6)	
O5	0.2143 (3)	0.4822 (3)	0.56750 (14)	0.0548 (5)	
H51	0.209 (7)	0.378 (3)	0.579 (3)	0.089 (13)*	
H52	0.202 (7)	0.529 (5)	0.6197 (17)	0.095 (14)*	
N1	0.3151 (3)	0.7460 (3)	0.44777 (14)	0.0440 (5)	
N2	−0.1135 (5)	0.9208 (4)	0.24863 (17)	0.0644 (7)	
C1	0.4205 (4)	0.6295 (3)	0.68336 (17)	0.0439 (6)	
C2	0.5205 (4)	0.6365 (3)	0.75573 (17)	0.0428 (6)	
C3	0.7119 (4)	0.6417 (4)	0.74021 (17)	0.0473 (6)	
H3	0.7806	0.6384	0.6845	0.057*	
C4	0.8014 (5)	0.6518 (4)	0.80646 (19)	0.0532 (7)	
H4	0.9292	0.6560	0.7954	0.064*	
C5	0.6993 (5)	0.6556 (4)	0.88983 (19)	0.0551 (7)	
C6	0.5098 (5)	0.6475 (4)	0.90521 (19)	0.0579 (8)	
H6	0.4424	0.6482	0.9609	0.069*	
C7	0.4202 (4)	0.6384 (4)	0.83915 (18)	0.0519 (7)	
H7	0.2927	0.6336	0.8503	0.062*	
C8	0.7904 (7)	0.6678 (6)	0.9615 (2)	0.0760 (11)	
H8	0.712 (5)	0.658 (4)	1.0166 (14)	0.062 (10)*	
C9	0.3345 (4)	0.8906 (3)	0.45861 (18)	0.0470 (6)	
H9	0.4275	0.8922	0.4899	0.056*	
C10	0.2234 (5)	1.0371 (4)	0.4256 (2)	0.0525 (7)	
H10	0.2408	1.1354	0.4348	0.063*	
C11	0.0859 (4)	1.0359 (3)	0.37870 (19)	0.0498 (7)	
H11	0.0102	1.1330	0.3550	0.060*	
C12	0.0623 (4)	0.8875 (3)	0.36748 (17)	0.0430 (6)	
C13	0.1776 (4)	0.7474 (3)	0.40382 (17)	0.0446 (6)	
H13	0.1590	0.6484	0.3976	0.054*	
C14	−0.1018 (4)	0.8756 (4)	0.32858 (19)	0.0495 (7)	
C15	0.0357 (6)	0.9707 (5)	0.1895 (2)	0.0743 (10)	
H15A	−0.0323	1.0752	0.1641	0.089*	
H15B	0.1251	0.9892	0.2214	0.089*	
C16	0.1546 (9)	0.8495 (9)	0.1215 (4)	0.141 (3)	
H16A	0.2588	0.8837	0.0896	0.212*	
H16B	0.0702	0.8423	0.0845	0.212*	
H16C	0.2126	0.7429	0.1459	0.212*	
C17	−0.2907 (7)	0.9202 (5)	0.2173 (3)	0.0779 (11)	
H17A	−0.4094	0.9678	0.2607	0.093*	
H17B	−0.3065	0.9890	0.1673	0.093*	
C18	−0.2717 (9)	0.7511 (6)	0.1959 (3)	0.1023 (16)	
H18A	−0.3799	0.7568	0.1692	0.153*	
H18B	−0.2757	0.6873	0.2468	0.153*	
H18C	−0.1470	0.6992	0.1576	0.153*	

### Atomic displacement parameters (Å^2^)

**Table:**

	*U*^11^	*U*^22^	*U*^33^	*U*^12^	*U*^13^	*U*^23^
Mn1	0.0401 (3)	0.0417 (3)	0.0406 (3)	−0.0139 (2)	−0.0145 (2)	0.0073 (2)
O1	0.0549 (12)	0.0520 (11)	0.0436 (10)	−0.0219 (9)	−0.0147 (9)	0.0043 (8)
O2	0.0448 (12)	0.0728 (15)	0.0577 (12)	−0.0197 (10)	−0.0128 (9)	−0.0024 (10)
O3	0.101 (2)	0.160 (3)	0.0737 (18)	−0.074 (2)	−0.0370 (16)	0.0082 (18)
O4	0.0537 (13)	0.0754 (15)	0.0651 (13)	−0.0343 (11)	−0.0159 (10)	0.0127 (11)
O5	0.0515 (12)	0.0615 (14)	0.0575 (13)	−0.0275 (10)	−0.0119 (10)	0.0061 (10)
N1	0.0426 (12)	0.0416 (12)	0.0481 (12)	−0.0131 (10)	−0.0153 (10)	0.0056 (9)
N2	0.0708 (18)	0.0782 (19)	0.0571 (15)	−0.0356 (15)	−0.0286 (13)	0.0165 (13)
C1	0.0459 (15)	0.0347 (13)	0.0483 (15)	−0.0089 (11)	−0.0142 (12)	0.0024 (10)
C2	0.0464 (14)	0.0369 (13)	0.0441 (14)	−0.0123 (11)	−0.0119 (11)	0.0024 (10)
C3	0.0479 (15)	0.0526 (16)	0.0414 (14)	−0.0197 (13)	−0.0060 (11)	0.0019 (11)
C4	0.0507 (16)	0.0617 (18)	0.0523 (16)	−0.0245 (14)	−0.0142 (13)	0.0034 (13)
C5	0.0603 (18)	0.0636 (19)	0.0451 (15)	−0.0244 (15)	−0.0147 (13)	0.0027 (13)
C6	0.0629 (19)	0.070 (2)	0.0395 (14)	−0.0251 (16)	−0.0068 (13)	0.0039 (13)
C7	0.0466 (16)	0.0579 (17)	0.0485 (15)	−0.0167 (13)	−0.0080 (12)	0.0025 (13)
C8	0.083 (3)	0.106 (3)	0.0521 (19)	−0.046 (2)	−0.0225 (18)	0.0047 (19)
C9	0.0475 (15)	0.0481 (15)	0.0506 (15)	−0.0200 (12)	−0.0165 (12)	0.0040 (12)
C10	0.0562 (17)	0.0428 (15)	0.0634 (18)	−0.0216 (13)	−0.0175 (14)	0.0072 (13)
C11	0.0514 (16)	0.0393 (14)	0.0582 (17)	−0.0141 (12)	−0.0174 (13)	0.0122 (12)
C12	0.0379 (13)	0.0458 (15)	0.0438 (14)	−0.0133 (11)	−0.0099 (10)	0.0061 (11)
C13	0.0452 (14)	0.0398 (14)	0.0511 (15)	−0.0149 (11)	−0.0163 (12)	0.0048 (11)
C14	0.0471 (15)	0.0512 (16)	0.0522 (16)	−0.0172 (13)	−0.0178 (12)	0.0099 (12)
C15	0.087 (3)	0.083 (3)	0.058 (2)	−0.035 (2)	−0.0189 (18)	0.0101 (18)
C16	0.115 (5)	0.184 (7)	0.129 (5)	−0.072 (5)	0.023 (4)	−0.060 (5)
C17	0.090 (3)	0.075 (2)	0.082 (3)	−0.033 (2)	−0.046 (2)	0.0196 (19)
C18	0.138 (4)	0.085 (3)	0.097 (3)	−0.039 (3)	−0.054 (3)	0.004 (2)

### Geometric parameters (Å, °)

**Table:**

Mn1—O1^i^	2.1596 (18)	C6—H6	0.9300
Mn1—O1	2.1596 (18)	C7—C6	1.377 (4)
Mn1—O5	2.207 (2)	C7—H7	0.9300
Mn1—O5^i^	2.207 (2)	C8—H8	0.971 (18)
Mn1—N1	2.283 (2)	C9—C10	1.375 (4)
Mn1—N1^i^	2.283 (2)	C9—H9	0.9300
O1—C1	1.262 (3)	C10—H10	0.9300
O2—C1	1.249 (3)	C11—C10	1.379 (4)
O3—C8	1.201 (5)	C11—H11	0.9300
O4—C14	1.232 (4)	C12—C11	1.385 (4)
O5—H51	0.924 (19)	C12—C13	1.375 (4)
O5—H52	0.926 (19)	C12—C14	1.500 (4)
N1—C9	1.337 (3)	C13—H13	0.9300
N1—C13	1.339 (3)	C15—C16	1.465 (7)
N2—C14	1.330 (4)	C15—H15A	0.9700
N2—C15	1.468 (5)	C15—H15B	0.9700
N2—C17	1.487 (4)	C16—H16A	0.9600
C2—C1	1.510 (4)	C16—H16B	0.9600
C2—C3	1.391 (4)	C16—H16C	0.9600
C2—C7	1.389 (4)	C17—C18	1.477 (6)
C3—C4	1.381 (4)	C17—H17A	0.9700
C3—H3	0.9300	C17—H17B	0.9700
C4—H4	0.9300	C18—H18A	0.9600
C5—C4	1.391 (4)	C18—H18B	0.9600
C5—C6	1.386 (5)	C18—H18C	0.9600
C5—C8	1.470 (4)		
			
O1^i^—Mn1—O1	180.000 (1)	O3—C8—C5	126.0 (4)
O1^i^—Mn1—O5	89.23 (8)	O3—C8—H8	121 (2)
O1—Mn1—O5	90.77 (8)	C5—C8—H8	113 (2)
O1^i^—Mn1—O5^i^	90.77 (8)	N1—C9—C10	123.1 (3)
O1—Mn1—O5^i^	89.23 (8)	N1—C9—H9	118.4
O1^i^—Mn1—N1^i^	92.23 (8)	C10—C9—H9	118.4
O1—Mn1—N1	92.23 (8)	C9—C10—C11	118.9 (3)
O1—Mn1—N1^i^	87.77 (8)	C9—C10—H10	120.6
O1^i^—Mn1—N1	87.77 (8)	C11—C10—H10	120.6
O5—Mn1—O5^i^	180.000 (1)	C10—C11—C12	118.8 (3)
O5—Mn1—N1^i^	92.95 (8)	C10—C11—H11	120.6
O5^i^—Mn1—N1^i^	87.05 (8)	C12—C11—H11	120.6
O5—Mn1—N1	87.05 (8)	C11—C12—C14	123.3 (2)
O5^i^—Mn1—N1	92.95 (8)	C13—C12—C11	118.3 (3)
N1^i^—Mn1—N1	180.00 (10)	C13—C12—C14	117.8 (2)
Mn1—O5—H52	101 (3)	N1—C13—C12	123.5 (3)
Mn1—O5—H51	118 (3)	N1—C13—H13	118.3
H52—O5—H51	106 (4)	C12—C13—H13	118.3
C1—O1—Mn1	126.76 (18)	O4—C14—N2	121.2 (3)
C9—N1—C13	117.3 (2)	O4—C14—C12	118.1 (3)
C9—N1—Mn1	124.02 (18)	N2—C14—C12	120.6 (3)
C13—N1—Mn1	118.65 (17)	N2—C15—H15A	108.7
C14—N2—C15	124.7 (3)	N2—C15—H15B	108.7
C14—N2—C17	117.3 (3)	C16—C15—N2	114.1 (4)
C15—N2—C17	118.0 (3)	C16—C15—H15A	108.7
O1—C1—C2	116.8 (2)	C16—C15—H15B	108.7
O2—C1—C2	117.8 (2)	H15A—C15—H15B	107.6
O2—C1—O1	125.4 (3)	C15—C16—H16A	109.5
C3—C2—C1	120.9 (2)	C15—C16—H16B	109.5
C7—C2—C1	119.8 (3)	C15—C16—H16C	109.5
C7—C2—C3	119.3 (3)	H16A—C16—H16B	109.5
C2—C3—H3	119.6	H16A—C16—H16C	109.5
C4—C3—C2	120.9 (3)	H16B—C16—H16C	109.5
C4—C3—H3	119.6	N2—C17—H17A	109.2
C3—C4—C5	119.6 (3)	N2—C17—H17B	109.2
C3—C4—H4	120.2	C18—C17—N2	111.8 (4)
C5—C4—H4	120.2	C18—C17—H17A	109.2
C4—C5—C8	120.7 (3)	C18—C17—H17B	109.2
C6—C5—C4	119.5 (3)	H17A—C17—H17B	107.9
C6—C5—C8	119.9 (3)	C17—C18—H18A	109.5
C5—C6—H6	119.5	C17—C18—H18B	109.5
C7—C6—C5	120.9 (3)	C17—C18—H18C	109.5
C7—C6—H6	119.5	H18A—C18—H18B	109.5
C2—C7—H7	120.1	H18A—C18—H18C	109.5
C6—C7—C2	119.9 (3)	H18B—C18—H18C	109.5
C6—C7—H7	120.1		
			
O5—Mn1—O1—C1	13.3 (2)	C3—C2—C1—O1	3.4 (4)
O5^i^—Mn1—O1—C1	−166.7 (2)	C7—C2—C1—O1	−176.9 (2)
N1^i^—Mn1—O1—C1	−79.6 (2)	C3—C2—C1—O2	−177.3 (3)
N1—Mn1—O1—C1	100.4 (2)	C7—C2—C1—O2	2.5 (4)
O1^i^—Mn1—N1—C9	−148.3 (2)	C1—C2—C3—C4	178.6 (3)
O1—Mn1—N1—C9	31.7 (2)	C7—C2—C3—C4	−1.2 (4)
O1^i^—Mn1—N1—C13	32.1 (2)	C1—C2—C7—C6	−178.9 (3)
O1—Mn1—N1—C13	−147.9 (2)	C3—C2—C7—C6	0.8 (4)
O5—Mn1—N1—C9	122.4 (2)	C2—C3—C4—C5	0.4 (5)
O5^i^—Mn1—N1—C9	−57.6 (2)	C6—C5—C4—C3	0.7 (5)
O5—Mn1—N1—C13	−57.2 (2)	C8—C5—C4—C3	−179.5 (3)
O5^i^—Mn1—N1—C13	122.8 (2)	C4—C5—C6—C7	−1.1 (5)
Mn1—O1—C1—O2	−29.8 (4)	C8—C5—C6—C7	179.1 (4)
Mn1—O1—C1—C2	149.49 (18)	C4—C5—C8—O3	6.7 (7)
Mn1—N1—C9—C10	179.1 (2)	C6—C5—C8—O3	−173.4 (4)
C13—N1—C9—C10	−1.2 (4)	C2—C7—C6—C5	0.3 (5)
Mn1—N1—C13—C12	−178.0 (2)	N1—C9—C10—C11	−0.3 (5)
C9—N1—C13—C12	2.3 (4)	C12—C11—C10—C9	0.9 (5)
C15—N2—C14—O4	−177.4 (3)	C13—C12—C11—C10	0.0 (4)
C17—N2—C14—O4	2.5 (5)	C14—C12—C11—C10	171.0 (3)
C15—N2—C14—C12	5.6 (5)	C11—C12—C13—N1	−1.7 (4)
C17—N2—C14—C12	−174.4 (3)	C14—C12—C13—N1	−173.2 (3)
C14—N2—C15—C16	108.8 (5)	C11—C12—C14—O4	−114.4 (3)
C17—N2—C15—C16	−71.1 (5)	C11—C12—C14—N2	62.7 (4)
C14—N2—C17—C18	−78.2 (5)	C13—C12—C14—O4	56.7 (4)
C15—N2—C17—C18	101.7 (4)	C13—C12—C14—N2	−126.3 (3)

Symmetry codes: (i) −*x*+1, −*y*+1, −*z*+1.

### Hydrogen-bond geometry (Å, °)

**Table:**

*D*—H···*A*	*D*—H	H···*A*	*D*···*A*	*D*—H···*A*
O5—H51···O4^ii^	0.92 (3)	1.87 (3)	2.775 (3)	165 (4)
O5—H52···O2	0.93 (3)	1.77 (4)	2.669 (3)	162 (4)

Symmetry codes: (ii) −*x*, −*y*+1, −*z*+1.

## References

[bb1] Antolini, L., Battaglia, L. P., Corradi, A. B., Marcotrigiano, G., Menabue, L., Pellacani, G. C. & Saladini, M. (1982). *Inorg. Chem.* **21**, 1391–1395.

[bb2] Bernstein, J., Davies, R. E., Shimoni, L. & Chang, N. L. (1995). *Angew. Chem. Int. Ed. Engl.* **34**, 1555–1573.

[bb3] Farrugia, L. J. (1997). *J. Appl. Cryst.* **30**, 565.

[bb4] Farrugia, L. J. (1999). *J. Appl. Cryst.* **32**, 837–838.

[bb5] Higashi, T. (1995). *ABSCOR* Rigaku Corporation, Tokyo, Japan.

[bb6] Hökelek, T., Budak, K. & Necefouglu, H. (1997). *Acta Cryst.* C**53**, 1049–1051.

[bb7] Hökelek, T., Çaylak, N. & Necefoğlu, H. (2007). *Acta Cryst.* E**63**, m2561–m2562.

[bb8] Hökelek, T., Çaylak, N. & Necefoğlu, H. (2008). *Acta Cryst.* E**64**, m505–m506. 10.1107/S1600536808005540 PMC2960864 21201885

[bb9] Hökelek, T. & Necefouglu, H. (1996). *Acta Cryst.* C**52**, 1128–1131.

[bb10] Hökelek, T. & Necefouglu, H. (1997). *Acta Cryst.* C**53**, 187–189.

[bb11] Hökelek, T. & Necefoğlu, H. (2007). *Acta Cryst.* E**63**, m821–m823.

[bb12] Hökelek, T., Necefouglu, H. & Balcı, M. (1995). *Acta Cryst.* C**51**, 2020–2023.

[bb13] Nadzhafov, G. N., Shnulin, A. N. & Mamedov, Kh. S. (1981). *Zh. Strukt. Khim.* **22**, 124–128.

[bb14] Rigaku (1998). *PROCESS-AUTO* Rigaku Corporation, Tokyo, Japan.

[bb15] Sheldrick, G. M. (2008). *Acta Cryst.* A**64**, 112–122. 10.1107/S0108767307043930 18156677

[bb16] Shnulin, A. N., Nadzhafov, G. N., Amiraslanov, I. R., Usubaliev, B. T. & Mamedov, Kh. S. (1981). *Koord. Khim.* **7**, 1409–1416.

